# Differential Effects of Constant Light and Dim Light at Night on the Circadian Control of Metabolism and Behavior

**DOI:** 10.3390/ijms21155478

**Published:** 2020-07-31

**Authors:** Valentina S. Rumanova, Monika Okuliarova, Michal Zeman

**Affiliations:** Department of Animal Physiology and Ethology, Faculty of Natural Sciences, Comenius University in Bratislava, Ilkovičova 6, 842 15 Bratislava, Slovakia; monika.okuliarova@uniba.sk (M.O.); michal.zeman@uniba.sk (M.Z.)

**Keywords:** circadian, rhythms, dim light at night, chronodisruption, melatonin, corticosterone, locomotor activity, behavior, hormones, metabolism

## Abstract

The disruption of circadian rhythms by environmental conditions can induce alterations in body homeostasis, from behavior to metabolism. The light:dark cycle is the most reliable environmental agent, which entrains circadian rhythms, although its credibility has decreased because of the extensive use of artificial light at night. Light pollution can compromise performance and health, but underlying mechanisms are not fully understood. The present review assesses the consequences induced by constant light (LL) in comparison with dim light at night (dLAN) on the circadian control of metabolism and behavior in rodents, since such an approach can identify the key mechanisms of chronodisruption. Data suggest that the effects of LL are more pronounced compared to dLAN and are directly related to the light level and duration of exposure. Dim LAN reduces nocturnal melatonin levels, similarly to LL, but the consequences on the rhythms of corticosterone and behavioral traits are not uniform and an improved quantification of the disrupted rhythms is needed. Metabolism is under strong circadian control and its disruption can lead to various pathologies. Moreover, metabolism is not only an output, but some metabolites and peripheral signal molecules can feedback on the circadian clockwork and either stabilize or amplify its desynchronization.

## 1. Introduction

Physiological and behavioral processes exert circadian rhythms, which have evolved to adapt organisms to daily environmental changes, such as the light:dark (LD) cycle. Circadian rhythms are generated endogenously, and as the term circadian (latin, circa – approximately, dien – day) suggests, their period is approximately 24 h in the absence of external cues.

The main molecular machinery generating circadian rhythms is the transcriptional–translational feedback loop consisting of positive (*bmal1* and *clock*) and negative (*per1*, *per2*, *per3* and *cry1*, *cry2*) elements in mammals [1,2]; these components represent the core of the molecular clocks. The proteins CLOCK and BMAL1 promote the transcription of *per* and *cry* genes and are expressed during the light phase in the central oscillator. In the cytoplasm, PER and CRY proteins are degraded by casein kinase 1ε [3] and AMP-activated protein kinase [4]. When both these enzymes are saturated, PER and CRY proteins form heterodimers that are translocated into the nucleus, interact with the CLOCK–BMAL1 complex and inhibit their own transcription [5]. The cycle, from the promotion to inhibition of clock genes transcription, lasts approximately 24 h. The molecular clock also contains accessory feedback loops, such as the one containing nuclear receptors, REV-ERB and retinoic acid-related orphan receptors competing to inhibit or activate the *bmal1* transcription, respectively [6,7]. 

Since the endogenous circadian rhythms do not exactly match the 24 h solar cycle, they must be synchronized (entrained) to the environmental cycles by external cues every day. The most potent synchronizing agent, also known as zeitgeber or entraining cue, is the LD cycle that is perceived by intrinsically photosensitive retinal ganglion cells containing photopigment melanopsin [8,9]. Consequently, the retinal signals are transmitted via the retinohypothalamic tract into the central oscillator located in the suprachiasmatic nuclei (SCN) of the hypothalamus [10]. The circadian system is hierarchically organized; the central pacemaker exerts its effects on peripheral oscillators via behavioral, neuronal and hormonal signals. Peripheral organs can also be entrained by other zeitgebers, such as food intake synchronizing the liver and pancreas, and these entraining cues can be more efficient than signals from the central oscillator [11,12,13]. 

Light mediates its non-visual effects via the SCN but can also directly affect different brain structures, which regulate certain physiological functions. Melanopsin-expressing intrinsically photosensitive retinal ganglion cells transmit information on lighting conditions directly to different brain areas, such as intergeniculate leaflet, the lateral geniculate nucleus of the thalamus [9,14,15], habenular nucleus, nucleus accumbens [9,15,16], medial amygdala, lateral hypothalamus and ventral preoptic area [9,15]. These structures can modulate sleep, mood and cognitive functions, heart rate and glucocorticoid levels [17].

Disruption of the molecular clockwork by environmental conditions can induce alterations in the body homeostasis, from behavior to metabolism. From the environmental cues, the LD cycle is of primary importance, since it has been very stable as life has evolved under LD conditions. However, over the last few decades, this environmental signal has lost its reliability because of the extensive use of artificial light at night [18,19]. The consequences of this new challenge on the performance and health of animals and humans are not fully understood and can be extremely important. Therefore, extensive research is needed to evaluate possible mechanisms and consequences of artificial light at night and predict the possible negative impacts on health and behavior. 

During a long history, people have been used to being exposed to high-intensity sunlight (~100,000 lx) during the day [20] and low-intensity moonlight (0.1–0.3 lx) during the full moon phase on a clear night [21]. In contrast, in recent years, people have experienced much lower intensity (400–600 lx) of lighting during the day and a higher illumination of 100–300 lx in the evening due to the lighting in offices and households [22]. Moreover, light-emitting devices (e.g., tablets, smartphones, computers) providing 30–50 lx of light are used at night [23]. Thus, the biological rhythms may not be well-tuned to the environmental cycles, with potential negative consequences on behavior, physiology and performance. The negative consequences of exposure to light at inappropriate times of the day can especially be observed in long-term shift-workers, who may suffer from metabolic diseases [24,25], cardiovascular diseases [26,27] and cancer [28,29]. The detrimental effects of artificial light at night are not observed only in humans but also in other ecosystems [30,31,32]. Moreover, recently, the rate of light pollution has been increasing rapidly due to urbanization and the introduction of efficient and cost-effective light-emitting diodes that accelerate the process of light pollution. The night-time illuminance in urban areas reaches 20 lx [33,34], and even 150 lx in some places [34]. 

The aim of the present review is to evaluate the effects of light pollution on chronodisruption of physiological processes and their control. We will assess the consequences induced by constant light (LL) in comparison to dim light at night (dLAN), since such an approach can shed light on underlying processes that are involved in mediating effects of chronodisruption in animal models. Constant light is a frequent study protocol for the induction of circadian disruption, and its consequences are described in this review, although these conditions are not usually present in real life. As a chronodisruption, we considered the damping of the amplitude of circadian rhythms, or even their elimination, as well as the disruption of rhythms in relation to cyclic environmental conditions (external misalignment) or their phases among each other (internal misalignment) [35]. For the purpose of this review, we characterized “constant light” as light exposure over a 24-h period and “dim light at night” as night-time light exposure not exceeding the intensity of 50 lx. 

This review included publications on the effects of LL and dLAN on the circadian control of physiological and behavioral processes and consequences of their disturbances on health. We searched the PubMed/MEDLINE databases for studies published in English between January 1990 and June 2020, using the following search terms: “constant light”, “artificial light at night”, “circadian rhythm”, “chronodisruption”, “clock gene”, “SCN”, “hormones”, “melatonin”, “pineal”, “glucocorticoid”, “metabolism”, “locomotor activity” and “obesity”. Relevant studies were assessed for inclusion by title and abstract, followed by a full-text review.

## 2. Circadian Hormonal Outputs

Probably the most known circadian hormonal outputs are melatonin and corticosterone (CORT), which display distinct circadian rhythmicity. Melatonin is synthesized in the pineal gland from an amino acid tryptophan, reaching its maximum during the dark phase. Therefore, melatonin is called “the hormone of darkness” [36]. The synthesis of melatonin is controlled by the multisynaptic pathway from the SCN [37]. As an internal zeitgeber, melatonin provides information about the length of the night through melatonin receptors, MT1 and MT2, which are widely distributed across the body [38]. For example, in pancreatic islets, MT1 receptors are highly expressed in the α-cells, while MT2 receptors predominate in the β-cells [39]. Pleiotropic effects of melatonin have been documented in a number of studies [38,40]. However, there are many controversial effects on metabolism, such as the improvement or worsening of glucose metabolism [41]. Melatonin receptors are also localized in the SCN [42,43] and melatonin has been proven to feedback to this master oscillator, adjusting its phase [43,44,45]. 

Corticosterone is a dominant glucocorticoid in rats, and its rhythmical release is under the control of the SCN [46], peaking before the onset of the active phase to prepare the organism for the upcoming stress events throughout the day [36]. One of the very important properties of CORT is its ability to set the phase of peripheral oscillators in many tissues [47,48]. This function is supported by the presence of glucocorticoid receptors in many tissues except for the SCN [47,49]. 

### 2.1. Melatonin and Corticosterone Levels Under Constant Light

Melatonin levels in mice vary greatly and many strains do not even produce this hormone [50], therefore, there are not much data on how LL influences melatonin levels. However, urinary 6-sulfatoxymelatonin is suppressed in melatonin-producing mice (CH3-HePas mice) during the subjective night in LL [51]. Unlike mice, rats produce melatonin, and its synthesis is highly sensitive to light exposure [52,53] (Table 1). Nocturnal, but not daytime, plasma melatonin levels are suppressed in LL, resulting in a loss of melatonin rhythm [54,55,56,57,58,59]. The same pattern is observed in urine 6-sulfatoxymelatonin concentrations [60]. 

Due to CORT rhythmicity, most studies measured its levels at the beginning and end of the inactive phase (the subjective day in constant conditions). In mice, LL was claimed to reduce CORT concentrations at the end of the subjective day [61,62,63]. However, this conclusion must be taken with a reservation because it is based on the two-points studies, which can miss shifts of acrophases of the rhythm. Therefore, studies measuring the 24 h profile of CORT in mice in LL are needed to elucidate the effects of constant light on circulating levels of this dominant glucocorticoid. The effects of LL on CORT in rats are diverse. In rats, its rhythm is abolished due to either the suppression of the peak [57,60] or the elevation of levels during the subjective day [59,64,65,66]. The effects do not seem to be strain-dependent since both changes are observed in Sprague–Dawley and Wistar rats, as shown in Table 1. Besides the altered plasma CORT, the adrenal CORT was elevated in the morning of the subjective day, resulting in the loss of diurnal variability which is in line with vanished differences between morning and afternoon in the adrenocorticotropic hormone that regulates the production of CORT in the adrenal gland [64]. The regulatory proteins of CORT synthesis and secretion, such as steroidogenic acute regulatory protein and steroidogenic factor 1, lost their daily variability on the mRNA and protein level. The loss of daily variability could be caused by the disturbed adrenal clock, since the CLOCK and BMAL1 regulate the production of steroidogenic acute regulatory protein [67]. Diminished signals from SCN, or the different distribution of food intake due to LL (described in the next section), could also impair the adrenal clock. The findings show that exposure to LL can influence the regulatory hypothalamo–pituitary–adrenal axis of CORT [64].

### 2.2. Melatonin and Corticosterone Levels Under Dim Light at Night

Similar to LL conditions, dLAN reduced nocturnal melatonin levels in rats [56,68,69]. The melatonin biosynthesis is highly sensitive to nocturnal light exposure, and even the illumination of 0.2 lx efficiently suppressed its high night-time concentration [56]. These suppressive effects on melatonin are comparable between LL and dLAN, regardless of the species or strain (Table 1 and Table 2). 

There is limited information about how dLAN influences CORT levels. The rhythm of CORT did not change in mice exposed to dLAN [62]. On the other hand, the cortisol peak was suppressed, and the rhythm was abolished in female Siberian hamsters [70]. In diurnal grass rats exposed to dLAN, CORT levels rose in the first half of the light phase [71] suggesting that diurnal and nocturnal species may differ in the response of this stress hormone to environmental lighting conditions. In rats exposed to dim red light at night, the CORT levels were reduced and phase-advanced compared to controls [68]. These studies suggest that there are interspecies and sex differences in the reaction of CORT to dLAN exposure (Table 2). Responses could also differ based on used lighting conditions, such as intensity, type of lighting or duration. To summarize, even though the responses of CORT to LL differ between species as well as strains, the loss of daily variability is often observed. However, there is not enough evidence on its rhythmicity in rodents exposed to LL or dLAN because in most experiments, CORT was measured only in two time-points of 24-h period. 

**Table 1:** Effects of constant light on behavior and physiology of mice and rats.

Species	Lighting Conditions	Food Intake	Body Mass	Locomotor Activity	Hormonal Rhythms	Metabolism	Ref.
C57Bl/6J mice M (10 wo)	≥180 lx (4 weeks)	Chow: No change in total food intake, SD = SN HFD: ↓ Total food intake, SD = SN	↑ Body mass	Reduced rhythmicity Lengthened period	Chow: CORT ZT1 = ZT11 HFD: ↓ CORT at ZT11	Endogenous glucose production, glucose infusion rate SD=SN ↑ Total RER, SD = SN ↓ Total energy expenditure, SD = SN	[61]
C57Bl/6J mice M (9–12 wo)	~580 lx (5 weeks)	↓ Total food intake	No change in body mass ↑ Fat mass and adipocyte size	Arrhythmic	N/A	↓ Glucose and FFA uptake ↑ Plasma FFA ↓ pAMPK, pCREB in brown adipose tissue	[72]
C57Bl/6J mice M (6 wo)	N/A (3 weeks)	No change in total food intake ↑ Daytime food intake	↑ Body mass No change in fat mass	N/A	N/A	Arrhythmic hepatic TAG ↑ Plasma TAG at ZT13 and ZT19 Plasma glucose phase-delayed Arrhythmic hepatic metabolic genes	[73]
C57Bl/6J mice M (10 wo)	N/A (10 weeks) HFD	No change in total food intake ↑ Daytime food intake	↑ Body mass ↑ Fat mass and adipocyte size	N/A	Melatonin suppressed ↑ Fasting insulin ↑ Insulin at ZT20 ↑ Leptin	Impaired glucose tolerance and insulin sensitivity ↑ Total plasma cholesterol ↑ Hepatic lipid accumulation Altered gene expression of metabolic genes	[74]
CD-1 mice M (6 wo)	100 lx (6 weeks)	No change in total food intake	No change in body mass	Reduced strength Lengthened period	Leptin ZT6=ZT18 ↓ TSH Free T3 ZT6=ZT18 ↑ Free T4	No change in fasting glucose and glucose tolerance	[75]
Swiss Webster mice M (8 wo)	150 lx (8 weeks)	No change in total food intake ↑ Daytime food intake	↑ Body mass ↑ Epididymal adiposity	No change in total activity Arrhythmic	CORT ZT7=ZT15	Impaired glucose tolerance at ZT11	[62]
C3H/HePas mice M (weaned)	N/A (8 weeks)	No change in total food intake ↑ Daytime food intake	↑ Body mass ↑ Fat mass Adipocyte hypertrophy	No change in total activity ↑ Daytime activity ↓ Night-time activity	↓ Night-time MT6s Insulin ZT2=ZT14	Insulin sensitivity and plasma glucose ZT2=ZT14 Hepatic expression of lipogenesis genes ZT2=ZT14 ↑ Plasma TAG at ZT14, hepatic TAG	[51]
HIP rats M (3 mo)	>100 lx (10 weeks)	N/A	↑ Body mass (tendency)	N/A	Melatonin suppressed	↑ Fasting plasma glucose at ZT2 ↓ Insulin secretion stimulated by β-cells at ZT2 ↓ β-cell mass and function	[54]
Long Evans rats M (5wo)	450 lx (17 days)	↓ Total food intake ↓ Total water intake	No change in body mass ↑ Visceral adiposity	Reduced rhythmicity Free running	↓ Night-time melatonin	N/A	[55]
Sprague-Dawley rat M (3 mo)	>100 lx (10 weeks)	N/A	No change in body mass	Arrhythmic	Melatonin suppressed	No changes in the glucose metabolism	[54]
Sprague-Dawley rat M (12 wo)	300 lx (3 weeks)	N/A	N/A	N/A	MT6s arrhythmic CORT arrhythmic Prolactin reversed rhythm	N/A	[60]
Sprague-Dawley rat M (5–6 wo)	200 lx (5 weeks)	N/A	N/A	N/A	↑ ACTH ↑ Adrenal CORT Plasma CORT ZT4=ZT12	N/A	[64]
Sprague-Dawley rat M (35–50 g)	300 lx (6 weeks)	No change in total food intake No change in total water intake	No change in body mass	N/A	Melatonin suppressed ↑ Total CORT, 2 peaks	Arrhythmic plasma lipids ↑ Glucose at ZT18	[56]
Sprague-Dawley rats Wistar rat Per1-LUC M (3 mo)	100 lx (10 weeks)	No change in total food intake	No change in body mass	Arrhythmic	Chow: Insulin arrhythmic HFD: ↑ Insulin, arrhythmic	Chow: No change in plasma glucose HFD: Hyperglycemia, ↓ amplitude of insulin secretory pulses	[76]
Wistar rat M (300–350 g)	150 lx (20 days)	No change in total food intake	No change in body mass	N/A	N/A	N/A	[77]
Wistar rat M, F (6 wo)	N/A (6 weeks)	N/A	↑ Body mass ↑ Fat mass	Arrhythmic	Melatonin arrhythmic CORT ZT1=ZT2 ↑ Fasting insulin at ZT0	↑ Hepatic and plasma TAG concentration at ZT0 ↑ Fasting glucose at ZT0 ↑ HOMA-IR	[57]
Wistar rat M, F (9 wo)	150 lx (4 weeks)	No change in total food intake	No change in body mass	N/A	No change in daytime melatonin ↑ T3/T4 (M) at ZT4-8 ↑ Insulin (F) at ZT4-8	No change in glucose ↓ Hepatic glycogen and glycogen phosphorylase at ZT4-8 ↑ Hepatic diacylglycerols and cholesteryl esters at ZT4-8	[58]
Wistar rat M (2 and 20 mo)	50–300 lx (30 days)	Arrhythmic (20 mo)	N/A	Arrhythmic	N/A	N/A	[78,79]

ACTH = adrenocorticotropic hormone. AMPK = AMP-activated protein kinase. CORT = corticosterone. CREB = cAMP response element-binding protein. F = female. FFA = free fatty acids. M = male. mo = months old. N/A = data were not available. MT6s = 6-sulfatoxymelatonin. RER = respiratory exchange ratio. SD = subjective day. SN = subjective night. wo = weeks old. T3 = triiodothyronine. T4 = thyroxine. TAG = triacylglycerols. TSH = thyroid-stimulating hormone. ZT = zeitgeber time.

**Table 2:** Effects of dim light at night (dLAN) on the behavior and physiology of mice and rats.

Species	Light Regimen	Food Intake	Body Mass	Locomotor Activity	Hormonal Rhythms	Metabolism	Ref.
C57Bl/6 mice PER2:luc M,F (10–12 wo)	12L:12DL (3 weeks) L: 150 lx DL: 5 lx	N/A	No change in body mass	WR activity: No change in total activity (after 3 weeks)	N/A	N/A	[80]
Swiss Webster mice F (8 wo)	16L:8DL (6 weeks) L: 150 lx DL: 5 lx	↑ Total food intake	↑ Body mass	No change in total locomotor activity	N/A	N/A	[81]
Swiss Webster mice M (>9 wo)	14L:10DL (2 weeks) L: 150 lx DL: 5 lx	No change in total food intake	↑ Body mass	No change in total locomotor activity	N/A	↑ RER ↓ Energy expenditure at ZT12–ZT16	[82]
Swiss Webster mice M,F (3 or 5 wo)	14L:10DL (6 weeks) L: ~130 lx DL: 5 lx	3 wo: ↑ Daytime food intake 5 wo: ↑ Daytime food intake (M)	3 wo: No change in body mass 5 wo: ↑ Body mass (M) ↑ gonadal fat	No change in total locomotor activity	N/A	3 wo: ↓ Fasted glucose (M), no change in glucose tolerance at ZT5 5 wo: No change in fasted glucose or tolerance at ZT5	[83]
Swiss Webster mice M (8 wo)	16L:8DL (8 weeks) L: 150 lx DL: 5 lx	No change in total food intake ↑ Daytime food intake	↑ Body mass ↑ Epidydimal fat	No change in total locomotor activity	No change in CORT	Impaired glucose tolerance at ZT11	[62]
Swiss Webster mice M (8 wo)	14L:10DL (5 days, 4 and 8 weeks) L: 150 lx DL: 5 lx	No change in total food intake ↑ Daytime food intake	↑ Body mass ↑ Epididymal fat	No change in total WR activity Disrupted WR rhythm in several animals (4 weeks)	Insulin ZT8=ZT14 (4 weeks)	No change in plasma glucose (4 weeks) Impaired glucose tolerance at ZT8 (4 and 8 weeks)	[84,85,86,87]
TALLYHO/ JngJ mice M (6 wo)	14L:10DL (4 and 8 weeks) L: 150 lx DL: 5 lx	N/A	↑ Body mass (not whole experiment)	N/A	N/A	↑ Daytime fasting glucose Impaired glucose tolerance Impaired insulin tolerance ↓ Survival of mice ↑ Number of mice with developed T2DM	[88]
Grass rats M (10 wo)	14L:10DL (3 weeks) L: 150 lx DL: 5 lx	N/A	No change in body mass No change in reproductive tissue mass	No change in total locomotor activity No change in rhythmicity	↑ CORT at ZT6	N/A	[71]
Sprague-Dawley rats M (35–50 g)	12L:12DL (6 weeks) L: 300 lx DL: 0.2 lx	No change in total food intake No change in total water intake	No change in body mass	N/A	Melatonin suppressed CORT phase advanced	No change in plasma lipids Normoglycemia	[56]
Sprague-Dawley rats M (3–4 wo)	12L:12DL (5 weeks) L: 300 lx DL: <10 lx (red light)	No change in total food intake No change in total water intake	No change in body mass	N/A	Melatonin suppressed ↓ CORT, phase advance ↓ Insulin, phase advance ↑ Leptin, 2 peaks	Total fatty acids suppressed ↑ Glucose, arterial pO2 and pCO2	[68]
Wistar rats M (~200–320 g)	12L:12DL (11 weeks) L: 150–200 lx DL: 5 lx	No change in total food intake ↓ Night-time food intake	No change in body mass No change in white adipose tissue	Dual rhythmicity	N/A	No change in glucose tolerance at ZT6 No change in total energy expenditure ↓ Night-time energy expenditure	[89]
Wistar rats M (18 wo)	12L:12DL (2 or 5 weeks) L: 150 lx DL: ~2 lx	N/A	N/A	N/A	Melatonin suppressed	N/A	[69]
SHR M (18 wo)	12L:12DL (2 or 5 weeks) L: 150 lx DL: ~2 lx	No change in total food intake	No change in body mass	N/A	↑ Daytime insulin No change in daytime leptin	No change in plasma metabolites ↑ Hepatic TAG (2 weeks) ↑ Hepatic *pparγ* (5 weeks) and expression of lipogenesis genes ↑ Adipose *pparα, pparγ* ↓ Cardiac *glut4*	[90]

CORT = corticosterone. DL = dim light phase. F = female. GLUT4 = glucose transporter 4. L = light phase. M = male. mo = months old. N/A = data were not available. pCO_2_ = partial pressure of carbon dioxide. pO_2_ = partial pressure of oxygen. PPAR = peroxisome proliferator-activated receptor. RER = respiratory exchange ratio. SHR = spontaneously hypertensive rats. T2DM = type 2 diabetes mellitus. TAG = triacylglycerols. wo = weeks old. WR = wheel-running. ZT = zeitgeber time.

## 3. Locomotor Activity, Food Intake and Body Mass

Locomotor activity is the reliable output of the circadian system and is often used to study changes in the central clock. Besides the locomotor activity, in this review, we also focus on alterations in feeding activity and body mass since both are closely related to metabolism.

### 3.1. Locomotor Activity Under Constant Light

**Figure 1:**
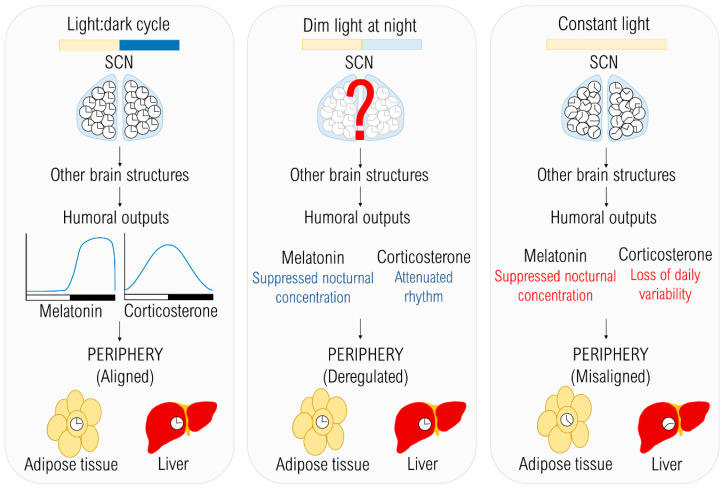
The effects of different lighting conditions on the temporal organization. The light:dark (LD) cycle provides an environmental cue entraining individual clocks in the suprachiasmatic nucleus (SCN), which are in synchrony with each other. Due to the entrained master pacemaker, humoral outputs are rhythmic and peripheral oscillators are also entrained to the LD cycle. In dim light at night (dLAN) conditions, the synchronizing cue is weaker than in LD, but exact mechanisms of how dLAN affects the central oscillator are not known yet. Melatonin and corticosterone rhythms are attenuated in rats (blue) and together with other internal signals can cause the deregulation of peripheral oscillators. In constant light (LL), which is a strong chronodisruptor, decoupling of individual clocks in the SCN occurs, resulting in disrupted rhythms of humoral outputs (red) and misalignment among peripheral clocks.

After LL exposure, the free-running rhythm with the lengthened period was described in locomotor activity, while mice exposed to LL for a longer time can display arrhythmic behavior (Table 1). Only one week of LL with an illuminance of 60 lx was not sufficient to induce the arrhythmicity of locomotor activity in mice, but one month initiated this change [91]. The manifestation of arrhythmicity can be accelerated by higher illumination. Mice under <10 lx LL displayed a lengthened period of their locomotor activity, and a similar effect was also observed under >200 lx, but some of the individuals became arrhythmic. This effect was even more apparent at higher intensities since 50% of mice were arrhythmic after exposure to >500 lx [92]. These findings were supported by other studies where the free-running rhythm with a lengthened period and the reduced strength of the rhythm appeared under LL of ≥180 and 100 lx [61,75], while the arrhythmicity occurred under much higher intensity (~580 lx) [72]. On the other hand, mice exposed to low illumination also developed arrhythmic locomotor activity without changes in total 24 h activity, but the experiment lasted two times longer [62]. The fact that not all mice became arrhythmic shows that the interindividual differences may also play an important role in the behavioral changes. In a study conducted with Per1:GFP transgenic mice, three types of behavioral changes were observed after LL exposure: a) arrhythmicity, b) period lengthening, and c) split rhythms [93]. Not only was locomotor activity varied among individuals, but the SCN neuronal activity changed and was in coincidence with alterations of behavior [61,93]. In arrhythmic mice, the clocks in SCN cells are desynchronized among each other, while they remain in synchrony in individuals with the longer free-running period. Splitting of the rhythm causes misalignment between SCN nuclei, the clocks of which are in antiphase [93]. Coincidence in locomotor and SCN neuronal activity indicates that the disruption of SCN has effects on locomotor activity and therefore, decoupling of the neurons in SCN could be one of the mechanisms behind the development of the arrhythmic locomotor activity in mice after exposure to LL (Figure 1). 

In rats, shortly after LL exposure, the rhythm of the locomotor activity became free-running with a longer period, but this rhythmicity was gradually lost, until, after approximately one month, the behavior was absolutely arrhythmic in all individuals [65,66,78], which is in agreement with other studies [54,57,76,78,79,94]. The displayed changes are comparable with changes in the behavior of mice; however, the interindividual differences are far more pronounced in mice. The free-running period observed in Long Evans rats exposed to LL for 17 days [55] could only represent the transition state after LL exposure [78]. The rhythms of *bmal1* and *rev-erbα* expression were abolished in the SCN of rats exposed to LL, but the expression of *per1* and *per2* genes maintained its rhythmicity [79]. Thus, the persistent rhythm in the expression of *per* genes was not sufficient to preserve daily changes in the locomotor activity. One of the mechanisms behind the alterations is the influence of melatonin on the locomotor activity. Melatonin increases the release of acetylcholine from the hypothalamic nucleus accumbens, and consequently, it leads to increased locomotor activity [95], suggesting that suppressed nocturnal melatonin levels are insufficient to stimulate acetylcholine release; therefore, the locomotor activity became reduced in Long Evans rats [55]. However, several mice strains do not produce melatonin [50], and even in melatonin producing and pigmented C3H/He mice, the interindividual differences in response to LL were described [96]. The locomotor activity might be re-entrained by other stimuli than light. For example, the rhythmic hormonal treatment with CORT and melatonin restored the rhythmicity of locomotor activity [57]. Another way to restore rhythmic behavior is through restricted feeding that synchronizes locomotor activity with the time of food access. Rats kept in LL with restricted feeding maintained the mRNA rhythmicity of *per2, bmal1* and *rev-erbα*, while the expression of *per1* was abolished in the SCN [79].

### 3.2. Locomotor Activity Under Dim Light at Night

The total amount of 24 h activity did not change after exposure to dLAN in Swiss Webster mice; however, there are insufficient data to demonstrate changes during the 24 h cycle [62,81,82,83,84,85,86]. The disrupted wheel-running rhythmicity was observed in four out of nine Swiss Webster mice that became arrhythmic [87] indicating the interindividual differences in sensitivity to circadian disruption. The wheel-running activity of C57Bl/6 mice was phase delayed after 5 days but not 3 weeks of dLAN [80], and these changes could be explained by a transition state. The rhythmicity of PER1 and PER2 was suppressed either in the SCN or the whole hypothalamus on both protein and mRNA levels, respectively [85]. However, the studies do not provide a detailed analysis of locomotor activity or the activity of single neurons in SCN; thus, further research is needed in this area. Long-term exposure (3 months) of more than 6 months old mice induced the attenuation of locomotor activity’s rhythm [97]. Higher light intensity (20 lx) increased daytime activity and phase-advanced the onset and offset of the locomotor activity [98]. Therefore, both age and illuminance level can modify the response to dLAN (Table 2). The total locomotor activity and rhythmicity under dLAN were comparable with LD conditions in diurnal grass rats [71]. The different response is described in Wistar rats that displayed dual rhythmicity of the locomotor activity that was not present in constant dim light conditions—therefore, the second peak developed specifically in dLAN conditions [89]. 

In contrast to LL, the arrhythmic locomotor activity was not recorded in both mice or rats kept in dLAN. Therefore, dLAN preserved the behavioral variation between the light and dim light phase, while the rhythmicity of the locomotor activity can be either unaffected, dampened or the second rhythm appears (Table 2).

### 3.3. Food Intake and Body Mass under Constant Light

Constant light did not influence total 24 h food intake [51,61,62,73,74,75], but the daily variation was suppressed mostly due to increased food consumption in mice during the subjective day [51,61,62,73,74]. On the other hand, total food intake was reduced in mice fed a high-fat diet [61]. These results suggest that the abnormal distribution of food intake can initiate elevated body weight gain [51,61,73,74] together with fat mass (Table 1) [51,72,74]. Even if the body mass is equal to the control group, the increase in fat mass and adipocyte size is observed [72]. 

The total food intake of rats in LL was mostly unaffected [56,58,76,77], but the distribution changed, causing the gradual loss of rhythmicity corresponding with changes in locomotor activity and neuronal activity in SCN [78]. The total food and water intake was reduced in Long Evans rats, probably due to strain differences or short-term exposure [55]. Despite this, the food efficiency was higher, therefore, rats gained more weight per gram of consumed food, although no change in body weight was noticed. Even though the distribution of food intake was altered, the body weight was not affected in rats [54,55,56,58,76,77], but still, the higher visceral adiposity was found [55], as indicated in Table 1. Only one study reported increased body weight and fat mass in Wistar rats after LL exposure [57].

### 3.4. Food Intake and Body Mass Under Dim Light at Night

In male Swiss Webster mice, dLAN increased the daytime food consumption, body mass and fat mass, while the total 24-h food intake was unaffected [62,81,82,83,84,85,86,87,88,99]. Female mice of the same strain exhibited an increased total food intake in dLAN [81], suggesting the existence of sex-dependent differences in response to dLAN. Food and water intake, body mass and fat mass were unaffected by dLAN in diurnal grass rats and nocturnal rats [56,68,71,89,90], although, in contrast to mice, the night-time food intake was reduced in rats [89]. Clearly, the interspecies differences can be observed in the responses of behavior and body weight to dLAN (Table 2). Regardless of lighting conditions, the loss of daily variability in feeding activity is often present, explaining at least partially the increased body weight and fat mass in mice, as well as in rats exposed to LL (Table 1).

## 4. Metabolic Effects

### 4.1. Glucose and Lipid Metabolism Under Constant Light

In mice, most studies have shown the loss of daily variation in metabolic measures due to LL conditions (Table 1). Insulin sensitivity was diminished during the subjective night, causing the loss of diurnal variation [51,61], which is in line with changes in insulin and glucose levels [51], as well as endogenous glucose production [61]. Glucose tolerance was impaired during the subjective day [62,74]. These data suggest that lower insulin sensitivity during the subjective night can advance the development of insulin resistance under LL. Another contributing factor could be the increased food intake during the daytime when the insulin sensitivity is lower and glucose tolerance is lower. It is known that feeding during an inappropriate phase of the day can misalign peripheral clocks, as well as the expression of metabolic genes and can disrupt energy balance, causing increased body weight gain [100]. CD-1 mice could be more resilient to LL, since, to our knowledge, it is the only strain without changes in glucose levels or glucose tolerance [75], or the potential effects of LL were not detected due to the chosen sampling time-point. 

Carbohydrates require less oxygen to be completely oxidized in comparison with fats [101,102], and the relative carbohydrate to fat oxidation ratio can be evaluated by the respiratory exchange ratio (RER) [103]. The higher total 24 h RER and a loss of variability was found in mice exposed to LL, indicating the preferential oxidation of carbohydrates over lipids [61]. Both high and low RER values are associated with obesity in mice [104,105,106]. Obese animals with decreased RER develop insulin resistance and they are probably not able to efficiently utilize carbohydrates and switch to fatty acid oxidation as the main energy source [104]. The lost diurnal pattern can be caused by changes in food intake, since higher RER values are in line with the loss of daily variability in the feeding activity [43]. In an agreement, another study showed that the light phase-fed mice displayed the elevated RER during the inactive phase [100]. 

On the other hand, the uptake of glucose and fatty acids was decreased by brown adipose tissue during the subjective day in mice after LL exposure, probably due to diminished β-adrenergic signaling, which also caused the suppressed expression of regulatory proteins, phosphorylated AMP-activated protein kinase and phosphorylated cAMP response element-binding protein [72]. AMP-activated protein kinase plays an important role in conserving and producing energy when there is a shortage of adenosine triphosphate (ATP). Therefore, it induces processes generating energy (e.g., fatty acid oxidation) and suppresses ATP-consuming processes (e.g., lipogenesis) [107,108]. The inefficient activity of brown adipose tissue and its lowered nutrient uptake can result in the lipid accumulation in the white adipose tissue [72]. After LL exposure, lost rhythmicity between subjective day and night was described in hepatic lipogenesis enzymes, fatty acid synthase and ATP citrate lyase, due to their elevated expression during the subjective day [51]. All these alterations lead to increased fat mass [51,62,72,74] and ectopic accumulation of lipids observed after exposure to LL [51,74]. Lipogenesis is regulated by transcription factors, sterol regulatory element-binding protein 1c and carbohydrate-responsive element-binding protein [109,110]; however, LL did not change their expression [51], suggesting the different mechanism of regulation. One of the regulatory proteins could be REV-ERBα which is involved in the control of lipid metabolism, especially in the suppression of lipogenesis, thus interconnecting metabolism with circadian clocks [111,112]. Suppressed expression of *rev-erb*α under LL conditions is in line with increased expression of lipogenic enzymes [51]. However, different results were observed in C57Bl6/J mice exposed to LL, in which hepatic triacylglycerols lost their rhythmicity, probably due to abolished rhythmicity and the suppression of the gene expression of lipogenic enzymes and transcriptional factors [73]. In this study, the rhythms in the expression of hepatic clock genes (*clock, cry1, per1* and *rorα*) were suppressed after exposure to LL, probably due to the altered distribution of food intake, which can efficiently entrain hepatic clocks. The response to LL differs between tissues, since the clock and metabolic genes in the liver were more affected than in the white adipose tissue [73]. These findings suggest the misalignment among peripheral organs, which can advance the development of metabolic diseases. However, the same strain of mice reacted differentially to the longer exposure of LL [74]. Changes occurred mainly during the subjective night and consisted of the increased hepatic accumulation and expression of metabolic genes and proteins involved in the control of lipogenesis and lipid metabolism [74]. 

Glucose levels were not affected in Sprague–Dawley rats during the subjective day in LL [54,56], but animals were hyperglycemic in the middle of the subjective night [56]. However, another study did not prove these changes since plasma glucose remained rhythmic under LL [76]. On the other hand, insulin lost its diurnal variation without changes in total 24 h mean values [76]. Strain can also play a role in different responses to LL (Table 1), since Wistar rats developed impaired glucose tolerance together with increased fasting glucose levels at the beginning of the subjective day [57] and increased insulin levels [57,58]. These changes in glucose and insulin concentrations increased the homeostatic model assessment of insulin resistance [57], which is used for establishing insulin resistance and β-cell function [113]. Another study did not find alterations in glucose—however, concentrations were measured in the middle of the light phase [58] in comparison to the onset of the light phase, as in the former study [57]. Nevertheless, hepatic glycogen and glycogen phosphorylase were reduced in rats exposed to LL [58], suggesting the decreased storage of glucose. Similar to mice, rats kept in LL probably utilized more carbohydrates, and therefore their deposition was suppressed and the hepatic accumulation of lipids was increased [57,58]. Plasma lipids also lost the daily variation in LL [56], probably due to different distribution of food intake. 

Constant light, in combination with other factors, can have even more deteriorating effects on insulin resistance (Table 1), since it was further aggravated in mice fed a high-fat diet while kept in LL [61]. Similar to mice, LL can also deteriorate the symptoms of other diseases in rats, such as diabetes. Impaired β-cell function and their increased apoptosis in rats kept in LL resulted in hyperglycemia and the loss of diurnal variation in insulin levels [76]. The diabetic strain of rats had accelerated development of hyperglycemia and suppressed insulin secretion from β-cells of pancreatic islets, probably due to increased apoptosis and decreased mass of β-cells during the subjective day [54].

### 4.2. Glucose and Lipid Metabolism Under Dim Light at Night

Dim light at night was not effective enough to change the daytime plasma glucose levels in mice [83,85], except in 3-week-old males that had reduced glucose levels during the light phase [83]. Glucose tolerance was impaired in adult mice [62,84,86] but not in young and adolescent animals exposed to dLAN, as shown in Table 2 [83]. The positive outcome of the studies was that disturbed glucose tolerance could be reversed by returning animals to dark nights [84] or by restricted feeding during the night [62]. Similarly to results from studies with LL, the RER was increased at the end of the dark phase and beginning of the light phase, suggesting the preferential oxidation of carbohydrates over lipids [82] and probably reflecting changes in food intake. Similarly to LL, the hepatic expression of *rev-erbα* was suppressed during the light phase in adult mice [85]; thus, the lipid metabolism can also be altered. However, altered expression of *rev-erbα* was not found in young or adolescent mice kept in dLAN [83]. Dim light at night differently influenced clock gene expression in white adipose tissue and liver, indicating the desynchronization among tissues [85]. 

In rats, levels of glucose and other metabolites were not affected by dLAN [56,89,90] and, contrary to mice (Table 2), glucose tolerance did not differ compared to control conditions during the light phase [89]. 

Similar to LL conditions, dLAN can also aggravate developed metabolic diseases in both mice and rats (Table 2). In diabetic mice, dLAN decreased survival and increased number of cases as well as accelerated development of glucose tolerance, insulin tolerance and increased fasting glucose levels [88]. In spontaneously hypertensive rats, the glucose levels were not affected by dLAN, but already high plasma insulin levels were even more aggravated after exposure to dLAN, suggesting that dLAN can deepen insulin resistance at least in this animal model [90]. This conclusion was supported by decreased gene expression of glucose transporter 4 in the left ventricle of the heart in spontaneously hypertensive rats exposed to dLAN. Moreover, the hepatic triacylglycerol content was elevated in rats after 2 but not 5 weeks of dLAN, and this could be explained by up-regulated gene expression of fatty acid synthase. The gene expression of peroxisome proliferator-activated receptor γ, the important transcription factor of genes involved in lipogenesis [114], has the opposite pattern to hepatic lipids [90,115], suggesting compensating effects of this nuclear transcriptional factor in lipid metabolism after 5 weeks of dLAN. The results from LL and dLAN conditions suggest that animals with early symptoms or developed diseases can be more vulnerable to the altered lighting conditions. 

## 5. Conclusions

Constant light disrupts endogenously generated circadian rhythms in physiology and behavior. Behavioral consequences of LL have usually been evaluated as rhythms in locomotor activity and consist of arrhythmia, changes in the free-running period and even “splitting the rhythm”, as shown convincingly in mice [93]. Alterations of these overt rhythms probably result from disrupted synchrony among cellular circadian oscillators localized in neurons of the SCN. Dim light at night disturbs a daily variability to a lesser extent than LL, and a phase-advanced or even dual rhythms were recorded in mice and rats, respectively [89,98]. Thus, it seems that dLAN does not affect the coupling of individual clocks localized in the SCN to the extent as constant light. The decoupling can relate to the intensity of dLAN and dose-dependent studies in this area are needed.

The attenuated or decoupled oscillations in SCN, due to dLAN can affect rhythmic feeding activity, which is usually limited to the active phase of the day (the night-time in nocturnal rodents). Food intake is a strong zeitgeber for peripheral tissues, especially the liver, and deregulated feeding cycles can misalign peripheral oscillators among each other and with the central oscillator [116]. After dLAN or LL exposure, the circadian timing system is not fully effective, and organisms lose their timing integrity, because physiological and behavioral rhythms are not in an appropriate phase or even eliminated. These conditions can have serious negative consequences for the brain and other body functions; it is expected to participate in the development and progress of many “diseases of civilization”, such as obesity, type 2 diabetes, cardiovascular and neural diseases and cancer. Therefore, consequences of dLAN should also be analyzed in relation to these pathologies, either in human studies or in animal models. 

The non-visual effects of light are not only mediated via the SCN but also other brain structures [9,14,15,16]. However, this area of research has not been covered sufficiently from the point of light pollution, even though it can play an important role in mediating the negative effects of LL and dLAN on physiology. Therefore, this gap should be filled in future studies.

Changes in metabolism seem to be more profound after exposure to LL in comparison with dLAN. Both conditions can deteriorate health status and be related to the level of light contamination, but a threshold which initiates these processes has not been established yet and should be determined. It is possible that there is no single value, but a continuum, which reflects huge interindividual and interspecies differences, nocturnality/diurnality and probably also a photoperiodic history of individuals. As indicated in Table 1, different levels of irradiance have been applied in experiments and these differences, together with the quality of light sources, can contribute to the high variability of obtained results. From the included studies, it is obvious that different rhythms lose their circadian “properties” at different levels of irradiance. This may reflect a different power of how the lighting conditions induce desynchronization among SCN neurons, as well as stabilizing rhythmic inputs from peripheral organs (behavioral and physiological rhythms). Generalization on the basis of one rhythm (locomotor activity) can be oversimplifying and therefore it is necessary to evaluate additional behavioral (drinking, feeding, response to aversive stimuli) and physiological (body temperature, cardiovascular rhythms, hormonal, metabolic and immune) parameters which can feedback to the SCN and stabilize its intrinsic property and circadian output rhythms. Moreover, there is a lack of studies exploring parameters of circadian rhythm such as mesor, acrophase or amplitude after dLAN exposure, because the usually employed two time-point studies cannot reveal complex changes in rhythmicity.

Clearly, more studies are needed to understand the effects of circadian disruption and links which result in the acceleration of pathophysiological processes. This understanding is important to elucidate the role of chronodisruption induced by artificial light as an influenceable factor in the development of diseases of civilization in humans.

## Abbreviations

AMPK	AMP-activated protein kinase
ATP	Adenosine triphosphate
BMAL1	Brain and muscle ARNT-like 1
CLOCK	Circadian locomotor output cycles kaput
CORT	Corticosterone
CREB	cAMP response element-binding protein
CRY	Cryptochrome
dLAN	Dim light at night
HOMA-IR	Homeostatic model assessment of insulin resistance
LD	Light:Dark
LL	Constant light
PER	Period
PPAR	Peroxisome proliferator-activated receptor
RER	Respiratory exchange ratio
ROR	Retinoic acid-related orphan receptors
SCN	Suprachiasmatic nuclei
SHR	Spontaneously hypertensive rats
